# 18F-FDG PET-CT Scans in Oncology Patients Treated with Hyaluronic Acid Filler: Not Always a Pitfall

**DOI:** 10.1155/2024/5559093

**Published:** 2024-03-27

**Authors:** Ilaria Proietti, Chiara Battilotti, Francesca Svara, Ersilia Tolino, Nicoletta Bernardini, Nevena Skroza, Luca Filippi, Concetta Potenza

**Affiliations:** ^1^Dermatology Unit “Daniele Innocenzi”, “A. Fiorini” Hospital, Via Firenze, 1, 04019 Terracina, Italy; ^2^Nuclear Medicine Unit, Department of Oncohaematology, Fondazione PTV Policlinico Tor Vergata University Hospital, Viale Oxford 81, 00133 Rome, Italy

## Abstract

The use of hyaluronic acid (HA) fillers in oncology patients undergoing PET-CT scans is a topic of debate due to potential interference with imaging accuracy. A 54-year-old female, postmelanoma metastasectomy in the parotid region with subsequent facial nerve palsy (FNP), received HA filler injections for facial symmetry and functional restoration. Follow-up PET-CT scans showed no interference or artifacts attributable to HA injection, allowing for accurate imaging results. This case suggests that HA fillers administered in oncology patients may not universally pose challenges or disrupt PET-CT imaging interpretation. Due to the possible false positives induced by fillers, the inclusion of aesthetic treatments in patients' anamnesis is a crucial step to accurately interpret PET-CT images. Although maintaining high level of caution in interpreting PET-CT results after filler injection is essential, our case emphasizes the safety of this procedure in oncology patients undergoing follow-up PET-CT scans.

## 1. Introduction

The use of HA fillers for addressing aesthetic and functional sequelae in oncology patients is garnering attention within the medical community. HA filler injection is becoming increasingly popular, with more than 4.8 million procedures performed in the US in 2022 alone [1]. This treatment minimizes wrinkles and fine lines, restores lost volume, and improves overall facial harmony with immediate results and minimal recovery time. HA is a polysaccharide naturally present in human dermis, which binds the collagen and elastic fibres to provide intercellular stability. HA injected combined with the body's natural HA binds water due to its hygroscopic nature and also induces new collagen formation. Depending on the degree of cross-linking, HA fillers induce soft-tissue augmentation which can last from several months up to 2 years. Compared to other forms of aesthetic intervention, HA fillers are temporary, less costly, ambulatory, and with low complication rates [2]. Despite their widespread use for cosmetic purposes, the utilization in oncology settings raises concerns about the potential impact on diagnostic imaging, including positron emission tomography-computed tomography (PET-CT) scans. 18F-FDG PET-CT imaging plays a pivotal role in oncology, providing valuable insights into disease progression and treatment response. Its high sensitivity and capability to identify areas with increased glucose metabolism allow the detection of lesions, sometimes undetectable by other imaging techniques. However, F18 fluorodeoxyglucose (FDG) is not a specific tumor marker, leading to potential false positives observed in approximately 6.5% of cases [3]. False positives can manifest in various conditions, primarily inflammation and infections [4], sometimes posing a considerable challenge to nuclear medicine specialists in distinguishing neoplastic from nonneoplastic metabolic activity. Specifically, HA filler is FDG avid and potentially leads to false positive results during staging and surveillance of cancer patients [5]. The increase in survival rates among oncology patients has led to a prolongation of patient's coexistence with long-term sequelae of cancer diagnosis and therapies. With more people with cancer living longer, improving patients' quality of life (QoL) becomes paramount. Dermatologic adverse events secondary to oncological therapy are common and negatively impact patients' QoL, affecting psychosocial well-being, body image, and social interactions [6]. This recognition highlights the significant role of aesthetic treatments in mitigating the physical and psychological consequences of cancer therapies, thereby contributing to patients' improved well-being [7]. Thanks to their biocompatibility and low immunogenicity, HA fillers are potentially suitable even in patients with immune system dysregulation or those undergoing immunotherapy. However, due to the slightly increased risk of hypersensitivity reactions in these patient categories, and the absence of extensive clinical studies exploring the interaction between HA fillers and the immune system, many specialists do not perform HA-based aesthetic treatments in patients with an overactive immune system [8]. Treatment choices need to be safe and compatible with ongoing anticancer therapies and not compromise oncologic outcomes. In this context, HA fillers are a safe and minimally invasive intervention effectively used to improve premature aging, correct atrophy from radiation therapy, address postsurgical asymmetry, and restore volume loss [9].

## 2. Case Presentation

**Figure 1:**
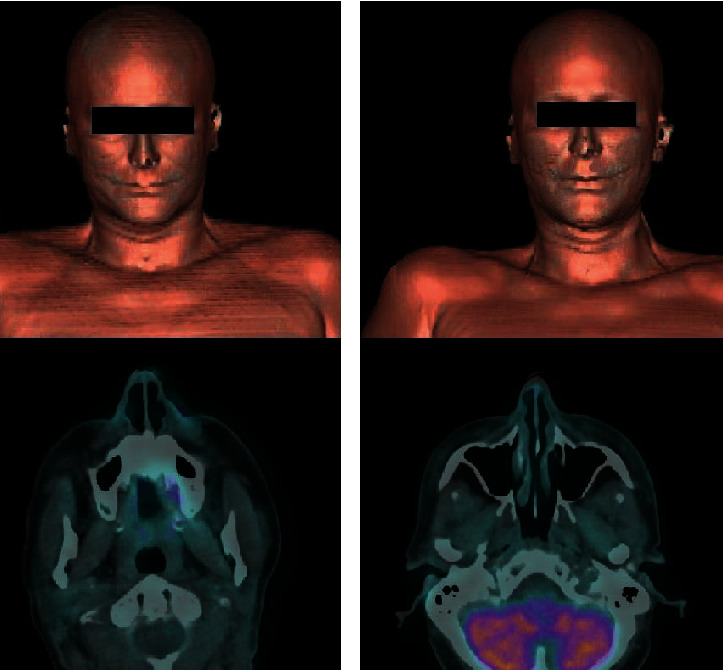
(a) 18F-FDG PET/CT: surface rendering (SR) of the skull (upper row) and corresponding fused axial PET/CT of the zygomatic region (lower row) before filler implant. (b) 18F-FDG PET/CT acquired during follow-up and after filler injection: SR of the skull (upper row) and fused axial PET/CT of the zygomatic region: no abnormal uptake, as a biomarker of inflammation, is evident with respect to the previous scan.

A melanoma 54-year-old female underwent removal surgery for metastasis in the parotid region and subsequent adjuvant therapy with BRAF/MEK inhibitors. Following surgical intervention, the patient experienced left-side FNP leading to difficulties in both alimentation and social interactions. Given the functional limitations impacting the patient's quality of life, a decision was made to address these concerns through injectable interventions. The therapeutic approach involved the administration of botulinum toxin (BoNT) to improve facial asymmetry and subsequently, after a period of 15 days, the use of HA injections at supraperiostial and subcutaneous levels to improve muscular movement and facial harmony [10]. The patient showed no adverse events to the injectables and drastically improved her quality of life. The patient underwent a follow-up 18F-FDG PET-CT scan approximately six months after the HA injection. No abnormal uptake is evident with respect to the previous PET-CT scan (Figure 1).

## 3. Discussion

Since its development in the late 20th century, PET-CT has become an indispensable tool in the oncology field, playing a crucial role in tumor staging, treatment evaluation, and surveillance [11]. With the increase in the 5-year survival rate for all cancers combined [12], there is an expected corresponding rise in the utilization of PET-CT imaging. Simultaneously, the higher survival rates lead to an increase in oncology patients experiencing sequalae from anticancer therapies. In fact, many cancer patients express discomfort with their appearance due to scarring or disfiguring surgeries. In this regard, the demand of aesthetic treatments, such as HA fillers, is growing, as they significantly improve patient's quality of life [9].

On PET-CT imaging, HA fillers exhibit FDG avidity, with several cases documented in the literature showing positive PET-CT scan results ranging from 2 weeks to 15 months following HA filler injections [13, 14]. Recently, Eitan et al. reported the case of a 49-year-old female who received HA filler injections to correct soft tissue defect following wide local excision of a low-grade angiosarcoma on the right cheek. Her PET-MRI 3 months later revealed low-grade hypermetabolism in the right maxillary region, leading to concerns of local recurrence [15]. The presence of fillers, commonly linked to local inflammatory responses, often leads to false positive PET findings suggestive of tumor recurrence or metastases potentially leading to unnecessary procedures and anxiety for patients [16]. In particular, HA filler demonstrates an increased FDG uptake also many months after injection and may resemble melanoma metastasis [16].

In oncology patients, fillers are regarded as safe; however, they are not devoid of side effects, which encompass injection site reactions, infections, and immediate or delayed hypersensitivity reactions. Specifically, granulomatous reactions occur between 0.04 and 0.3% of filler injection cases, with immunotherapy being identified as a risk factor for their occurrence. Checkpoint inhibitors appear to act as a trigger for the formation of foreign bodies against fillers, also many years after HA injection [8]. Conversely. there is only one reported case of a granulomatous/sarcoid-like reaction due to silicone filler in a patient receiving BRAF/MEK inhibitors [17]. Among melanoma therapies, BRAF/MEK inhibitors appear to be safer; however, patients must be informed about this adverse reaction, which, although very rare, can occur even many years following the filler injection. In our case, the patient showed no adverse reaction to the HA filler over a 24-month follow-up period.

The absence of PET-avidity post filler injections in this specific case challenges the conventionally held notion regarding the consistent interference of fillers in PET accuracy. While our case presents compelling findings, further comprehensive investigations involving larger cohorts and diverse filler compositions are warranted.

## 4. Conclusion

Radiologists and clinicians should exercise caution and maintain awareness of potential false-positive PET findings in oncology patients who underwent filler injections. However, our case suggests a potential scenario where certain filler compositions or injection techniques may not universally affect PET imaging interpretation. Studies with large samples and different filler compositions and injection techniques are necessary to evaluate the long-term safety and efficacy of injectable treatments in oncology patients, as well as their impact on PET-CT imaging results. Aesthetic interventions, when carefully integrate into comprehensive patient care, not only enhance patients' quality of life but also show their safety profile in the oncology setting. Collecting a comprehensive patient anamnesis, including any history of filler treatments, is mandatory to correctly interpret PET-CT images. This approach is essential for avoiding misinterpretations that could lead to unnecessary patient anxiety or interventions, thereby promoting more accurate and reliable diagnostic outcomes.

## Data Availability

The data that support the findings of this study are available from the corresponding author upon reasonable request.
